# Ecological drivers of dog heartworm transmission in California

**DOI:** 10.1186/s13071-022-05526-x

**Published:** 2022-10-23

**Authors:** Lisa I. Couper, Erin A. Mordecai

**Affiliations:** Department of Biology, Stanford University, Stanford, CA 94305 USA

**Keywords:** Heartworm, Vector, Mosquito, Land cover, Climate, Disease ecology, Transmission

## Abstract

**Background:**

Effectively controlling heartworm disease—a major parasitic disease threatening animal health in the US and globally—requires understanding the local ecology of mosquito vectors involved in transmission. However, the key vector species in a given region are often unknown and challenging to identify. Here we investigate (i) the key vector species associated with transmission of the parasite, *Dirofilaria immitis*, in California and (ii) the climate and land cover drivers of vector presence.

**Methods:**

To identify key mosquito vectors involved in transmission, we incorporated long-term, finely resolved mosquito surveillance data and dog heartworm case data in a statistical modeling approach (fixed-effects regression) that rigorously controls for other unobserved drivers of heartworm cases. We then used a flexible machine learning approach (gradient boosted machines) to identify the climate and land cover variables associated with the presence of each species.

**Results:**

We found significant, regionally specific, positive associations between dog heartworm cases and the abundance of four vector species: *Aedes aegypti* (Central California), *Ae. albopictus* (Southern California), *Ae. sierrensis* (Central California), and *Culiseta incidens* (Northern and Central California). The proportion of developed land cover was one of the most important ecological variables predicting the presence or absence of the putative vector species.

**Conclusion:**

Our results implicate three previously under-recognized vectors of dog heartworm transmission in California and indicate the land cover types in which each putative vector species is commonly found. Efforts to target these species could prioritize surveillance in these land cover types (e.g. near human dwellings in less urbanized settings for *Ae. albopictus* and *Cs. incidens*) but further investigation on the natural infection prevalence and host-biting rates of these species, as well as the other local vectors, is needed.

**Graphical Abstract:**

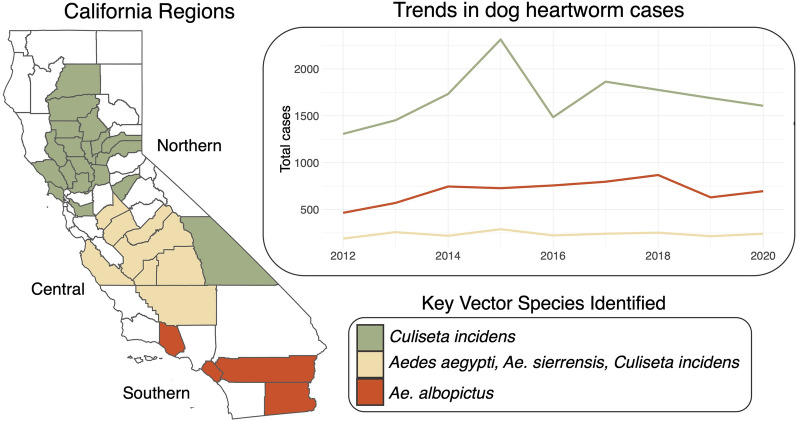

**Supplementary Information:**

The online version contains supplementary material available at 10.1186/s13071-022-05526-x.

## Background

Heartworm is a globally distributed, mosquito-borne disease of high veterinary and economic importance. The parasitic roundworm, *Dirofilaria immitis*, can cause severe organ damage and death in a range of companion and wild animals including canids (e.g. dogs, coyotes), felids (e.g. cats, tigers), mustelids (e.g. ferrets, otters), and pinnipeds (e.g. sea lions, seals) [1]. *Dirofilaria immitis* can also cause pulmonary dirofilariasis in humans, making heartworm a zoonotic disease of concern to public health [2]. Heartworm preventatives are highly effective, yet they are prohibitively expensive for many pet owners and drug-resistant pathogen strains have recently emerged [1, 3, 4]. As a result, roughly 1–12.5% of domestic dogs in the US are estimated to be infected [5, 6]. More effective disease prevention and control require a better understanding of the drivers of disease transmission.

Heartworm transmission involves several developmental stages of *D. immitis* in both the mosquito vector and definitive mammalian host [7, 8]. Mosquitoes ingest microfilariae (L1) during blood-feeding on an infected host, which develop within the mosquito to reach the infective third larval stage (L3). This process takes approximately 8–30 days depending on temperature, with faster parasite development at higher temperatures [9]. During the next mosquito blood meal, the infective stage enters the bite wound of a susceptible host where it completes development. Between 6 to 9 months after host infection, mature *D. immitis* release microfilariae, which can be detected in the host blood stream.

Mosquito vector presence was identified as the greatest risk factor of dog heartworm prevalence in the US [10], making vector control necessary for disease prevention. At least 25 species from five genera (*Aedes, Anopheles, Culex, Culiseta*, and *Psorophora*) are potential vectors based on natural infections with L3 filariids in collected individuals, and many additional species have demonstrated competence in laboratory assessments [11]. However, determining which mosquito species are important drivers of transmission in a given region is challenging as this depends not only on physiological competence, but also vector abundance and propensity for feeding on host species [12, 13]. Furthermore, within-species variation in vector competence and host-biting rates have been observed, leading to variation in the key vector species involved in transmission between different regions and vector communities in the US [14–17]. As the most effective vector control measures can vary by species, preventing transmission requires understanding the local mosquito ecology and disease transmission cycle [11].

Climate and land cover factors have also been identified as important drivers of dog heartworm transmission, primarily given their impact on vector distributions and abundance [10]. Temperature impacts mosquito development rates, survival, and reproduction, as well as pathogen development rates within the mosquito, while moisture conditions govern the availability of mosquito breeding habitat [18, 19]. However, identifying the specific aspects of temperature and precipitation that most strongly impact the abundance of a particular species remains challenging [19–22]. Accordingly, several temperature variables such as maximum, minimum, mean, and diurnal temperature and precipitation variables at various lags have been proposed as drivers of vector presence and dog heartworm transmission [10, 24–27]. Similarly, numerous land cover variables such as the percent of forest, wetland, or impervious cover in the region have been proposed given their influence on mosquito and host abundance and mosquito-host contact rates [10, 16, 17, 25, 28]. Furthermore, each of the potential vector species may respond differently to these ecological features. For example, *Ae. vexans* are typically found at higher abundance in partially shaded floodwaters and woodlands, while *Cx. quinquefasciatus* are found at higher abundance in more developed landscapes [11]. Identifying which aspects of climate and land cover are most strongly associated with the abundance of key vector species can aid in determining when and where to target vector control and heartworm preventative efforts.

**Fig. 1:**
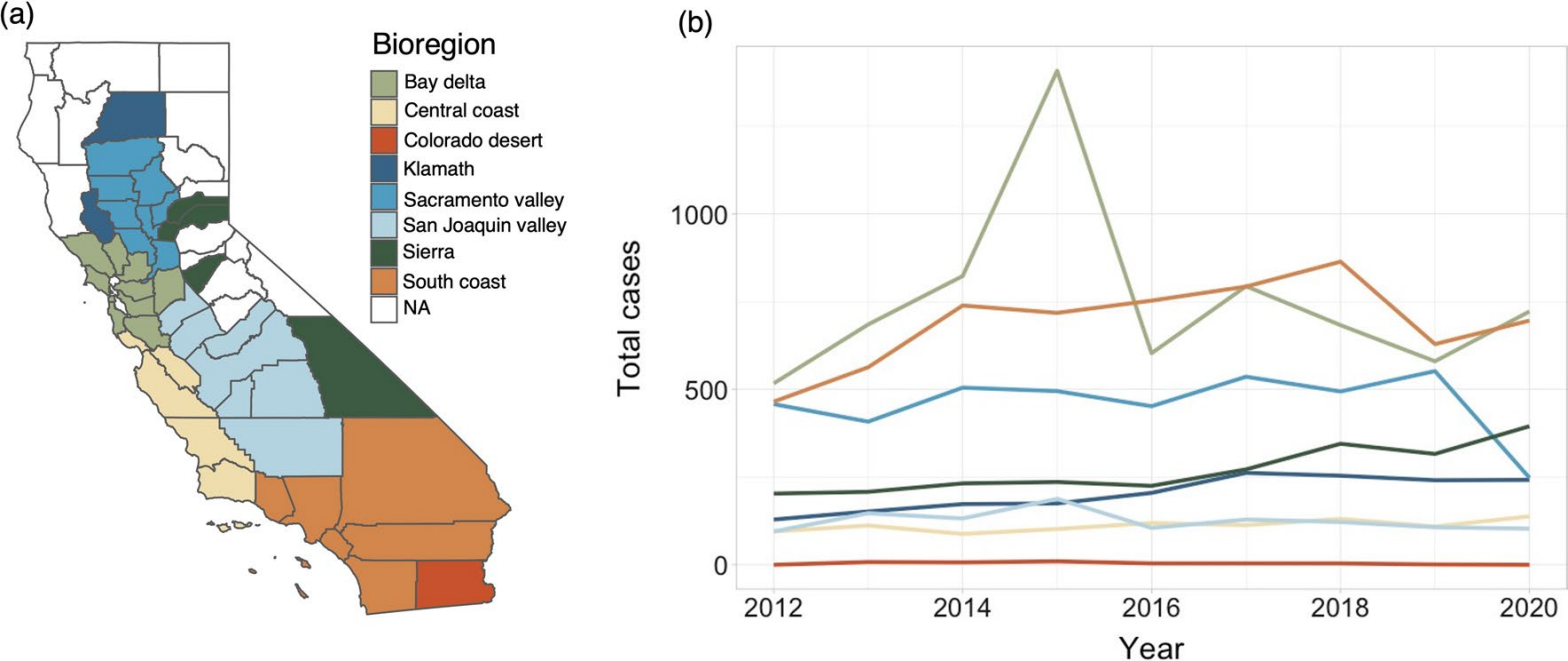
**a** California bioregions as developed by the Interagency Natural Areas Coordinating Committee, based on distinct physiographic properties and ecological communities. Counties not included here (shown in white) were those without reported mosquito surveillance data. **b** Trends in dog heartworm cases, shown here as the total number of reported cases in a given year, from 2012 to 2020 for each California bioregion

The complexity of dog heartworm transmission, including the many interacting and nonlinear biotic and abiotic factors, and lags in infection and transmission, as well as the lack of pathogen surveillance in mosquitoes, make determining the drivers of transmission challenging. Here, we investigate the ecological drivers of dog heartworm transmission in California—an ideal region for this investigation as the state harbors nine putative vector species (Table 1), contains high ecological diversity (Fig. 1a), and has experienced recent increases in dog heartworm cases in several regions (Fig. 1b). Furthermore, long-term mosquito surveillance records have been collected by vector control districts across the state, and dog heartworm case counts have been collected for the past decade, making it possible to infer relationships between vector populations and transmission. We leverage these rich data sources to investigate the biotic and abiotic drivers of dog heartworm transmission in the state. We analyze Northern, Central, and Southern California separately as variations in vector competence and dog-biting rates have been found between these regions (Table 1).

**Table 1:** Prior information on putative vector species

	Vector competence	Abundance	Dog-biting rates
*Ae. aegypti*	- Some strains found to be capable of infection during laboratory trials, other strains resistant or highly incompetent [38–42] *- D. immitis* found in field-collected strains in Florida [43] and Argentina (non-infective stage; 64) - Species presence negatively associated with dog heartworm transmission in US [25]	Found in nearly all bioregions but at relatively low abundance	High rates of dog-biting found in field-collected individuals from TX [45], but unknown in CA
*Ae. albopictus*	*- D. immitis* identified in field-collected adults in FL [46], GA [47], LA [48], NC [17], and OK [16] - Low and high (strain-specific) vector competence found in lab and field studies [17, 49–51] - Species presence negatively associated with dog heartworm transmission in US [25]	Found only in the Klamath and South Coast bioregion and at low abundance	Moderate to high rates of dog-biting in Eastern and Midwestern US [52–55], but unknown in CA
*Ae. sierrensis*	*- D. immitis* infection in field-collected adults have been found at both low and high rates in Northern and Southern CA [56–58] - Species presence positively associated with dog heartworm transmission in Northern CA [59] and US [25]	Found in nearly all bioregions but at relatively low abundance	Low to moderate rates of dog-biting on field-collected and experimental adults in CA [56, 60–62]
*Ae. vexans*	- Prevalence of *D. immitis* in field-collected individuals was found to be high in Northern CA, AR, and MN [56, 63, 64], and moderate in Southern CA [65] - High vector competence found in laboratory studies using adults from MN [66]	Found in nearly all bioregions, at low to moderate abundance	High rates of dog-biting in experimental populations in CA [56] and field-collected populations in WI [67]
*An. freeborni*	- High vector competence found during lab studies on collections from Northern CA [68, 69] and SC [70] - Moderate rates of *D. immitis* prevalence in field-collected adults in Northern CA [58]	Found in nearly all bioregions. Abundant in Northern, but not Southern CA	Low to moderate rates of dog-biting in field-collected adults in Northern CA [60, 61]
*Cs. incidens*	- Lab studies found low [68], moderate [71], and high [57] vector competence for adults from CA *- D. immitis* found in field-collected adults in Southern [65] but not Northern [68] CA	Found in nearly all bioregions, at low to moderate abundance	Low rates of dog-biting found in natural populations from Northern CA [61], but found to readily bite dogs during experimental exposures with adults from Northern and Southern CA [57, 68]
*Cs. inornata*	- Low vector competence found in lab studies using adults from Northern CA [68] - Naturally infected at moderate rates in Southern CA [65] and high rates in AR [63]	Found in nearly all bioregions, but at relatively low abundance	Low rates of dog-biting in Northern CA [61]
*Cx. quinquefasciatus*	- High rates of infection in field-collected adults found in AR [63], low rates of infection with non-infective stage found in LA [72] and AL [73] - Low vector competence in laboratory experiments using adults from Asia [41, 50], Haiti [74], and Brazil [14, 75] - Species presence positively associated with dog heartworm transmission in US [25]	Found in nearly all bioregions. Abundant in Southern CA	Low to moderate rates of natural dog-biting in Southern CA [76, 77]
*Cx. tarsalis*	*- D. immitis* prevalence in field-collected adults was low in Northern CA [58], but high in Southern CA [65] - Low vector competence found in laboratory studies using adults from CA [68] and MN [66]	Found in all bioregions at high abundance	Low to moderate rates of natural dog-biting in Northern CA [60, 61] and moderate rates in Southern CA [76]

Prior information on vector competence, local abundance, and propensity for dog-biting for the nine putative vector species of dog heartworm in California. States are referenced using two-letter state abbreviations (e.g. AR = Arkansas)

Specifically, we ask: (i) What are the primary vector species associated with dog heartworm transmission in Northern, Central, and Southern California? (ii) What are the key ecological drivers of vector presence for these species? For the first investigation, we use a statistical modeling approach (fixed-effects regression) that explicitly accounts for unobserved variation in dog heartworm cases between different biological regions and years to identify causal relationships between vector abundance and cases. For the second investigation, we use a flexible machine learning approach (gradient boosted machines) that can handle complex and collinear relationships between predictors and response variables and incorporate numerous climate and land cover variables at varying lags and scales to comprehensively assess the impact of climate and land cover on mosquito presence.

## Methods

### Dog heartworm case data

We used publicly available dog heartworm case data compiled by the Companion Animal Parasite Council [29]. These data are based on reports from IDEXX Laboratories, ANTECH Diagnostics, and Banfield Pet Hospital. Data include the number of dog heartworm antigen tests performed and the number of positive test results at the county and monthly scale from 2012 to the present. Antigen tests are typically performed at annual veterinary visits, but the CAPC notes the actual number of tests conducted is likely greater than their reports. The antigen tests have nearly 100% specificity and can detect sexually mature, adult female *D. immitis* from the blood of infected dogs starting at 5 months post-infection [30]. Infected dogs are typically treated and will test negative for antigens in subsequent years, meaning positive test results likely capture infection within the prior year. The reported county for a given test is based on the location of the veterinary clinic and thus could differ from the county in which the dog was infected.

### Mosquito data

We considered all mosquito species in California that are potential vectors of dog heartworm [11]: *Aedes aegypti, Ae. albopictus, Ae. sierrensis, Ae. vexans, Anopheles freeborni, Culiseta incidens, Cu. inornata, Culex quinquefasciatus*, and *Cx. tarsalis*. Surveillance data for these species were provided by the California Vectorborne Disease Surveillance Program (CalSurv), which represents over 60 California mosquito and vector control agencies, the California Department of Public Health, and the Davis Arbovirus Research and Training Lab at the University of California, Davis. We subset the available data to include only abundance records for adult females collected in a single trap night using gravid, oviposition, ${CO}_{2}$-baited, light, or BG sentinel traps or resting boxes (i.e. we excluded estimates from multiple collection nights or rare trap types). Furthermore, we removed any observation from vector control districts contributing fewer than five total records to minimize the effect of variation in surveillance methods and species identification. From these data, we calculated the average mosquito species abundance in a given county and year.

### Climate data

We used publicly available, modeled climate data from the NOAA Physical Sciences Laboratory (https://psl.noaa.gov). We obtained daily maximum and minimum land surface temperature and precipitation data for the period of 1979–2021 at a 0.5° × 0.5° spatial resolution. We use the climate data associated with the closest latitude and longitude for each mosquito surveillance site (*n* = 31,389 unique surveillance sites). From these data, we calculated several climate variables capturing temperature and precipitation conditions including the minimum, maximum, and mean (the average of the maximum and minimum) temperature, the diurnal temperature range (the difference in the maximum and minimum temperature), and total precipitation. We calculated these variables at varying time periods and lags, as in Skaff et al. [31]. This included daily, weekly, monthly, and quarterly variables at one, two, or three time steps prior to the date of mosquito collection (e.g. one, two, or three days prior to the surveillance record date for the daily climatic variables).

### Land cover data

We used the publicly available National Land Cover Database (NLCD) from the Multi-Resolution Land Characteristics consortium (https://www.mrlc.gov/data). Land cover data are available at 30-m resolution for the years: 2001, 2004, 2006, 2008, 2011, 2013, 2016, and 2019. From these data, we calculated the proportion coverage of the following classes: forest (evergreen, deciduous, or mixed), wetland, herbaceous, shrubland, low intensity developed (a mixture of constructed and vegetative materials), and high intensity developed (80–100% impervious cover). We calculated each of these at both 100- and 1000-m buffers around each surveillance site. Variables were calculated using the *raster* package in R v 4.0.2 [32]. For each surveillance record, we used the NLCD data from the given surveillance year or the closest preceding year.

### Dog density and human socioeconomic data

We used publicly available, annual, county-level data from the US Census Bureau (USCB) to estimate dog density (https://www.census.gov/programs-surveys/acs/data.html). Specifically, we used the estimated number of housing units, multiplied by 0.614—the average number of dogs per household [33]—divided by the county area ${(mi}^{2})$. While the densities of other hosts such as coyotes and other wild canids are putative drivers of transmission, there are no reliable, comprehensive, spatially and temporally resolved data on these species. We also obtained annual, county-level median household income data from the USCB, as prior studies have found lower dog heartworm cases associated with higher household income because of increased heartworm preventative compliance [10, 34].

### California bioregions

To delineate different regions of California based on ecological similarity, we used the 10 bioregions developed by the Interagency Natural Areas Coordinating Committee. These bioregions were determined based on distinctions in physiographic properties and ecological communities [35]. For counties that overlapped multiple bioregions, we selected the bioregion in which the largest portion of the human population resided, as this likely represented where dog heartworm cases were reported (Fig. 1a).

### Data analysis: identifying key vector species

To identify key vector species associated with dog heartworm transmission, we used a least squares dummy variable (or fixed effects) regression approach—a statistical modeling approach used to isolate potential causal relationships in settings where randomized experiments are infeasible [36]. We used both bioregion and year as dummy variables to control for any unobserved heterogeneity that might influence dog heartworm cases in a particular bioregion across all years (e.g. geographic features, number of veterinary clinics) or influence cases in all bioregions in a given year (e.g. an influx of shelter dogs to the state due to natural disaster [37], higher case reporting). Using these models, we included the abundance of each putative vector species as a predictor. Specifically, for each species, we used adult female abundance at a 1-year lag (to account for the lag between transmission and potential case detection) averaged across all traps within the county. We assessed the multicollinearity of these predictors by calculating the variance inflation factor (VIF). None of the mosquito predictors had a VIF value > 3; thus, we did not exclude any species from the model (Additional file 1: Figure S1). As host population density and socioeconomic status can impact case reporting [10, 25], we also included county-level dog population density and median household income as predictors, each at a 1-year lag. As broad spatial variation in vector abundance, competence, and dog-biting rates has been observed in California (Table 1), we ran separate models for Northern, Central, and Southern California. We classified the Bay Delta, Klamath, Sierra, and Sacramento Valley bioregions as Northern California, the Central Coast and San Joaquin Valley bioregions as Central California, and the South Coast and Colorado Desert bioregions as Southern California (Fig. 1a).

### Data analysis: ecological drivers of vector presence

To identify the key climate and land cover predictors of each of the nine putative vector species, we used a gradient boosted machine (GBM) approach implemented using the *XGBoost* package in R [78]. Briefly, gradient boosting is a supervised machine learning approach in which regression or classification trees is sequentially built from the prediction errors of the prior tree. GBM algorithms allow for complex, nonlinear relationships among predictor and outcome variables and collinearity between predictors, making them well suited for this analysis. Extreme gradient boosting is a scalable and efficient GBM implementation that minimizes overfitting and has been shown to achieve high predictive accuracy [79]. Here, we developed an XGboost classification model predicting the presence or absence of each putative vector species (specifically an adult female of that species in a single night at a single trap). Absences here refer to a specific surveillance site and day where a mosquito was found, but not of the given species (i.e. ‘true’ absences rather than pseudo-absences). All climate and land cover predictors described above (*n* = 78; see “Methods”: “Climate data,” and “Land cover data”) were initially considered as predictors for the model. However, as including many collinear variables can minimize interpretability, we removed any predictor with *a* > 0.90 pairwise correlation with another predictor (*n* = 31) and preserved those with the greatest biological relevance (*n* = 47; Additional file 3: Table S1). Furthermore, we log-transformed any highly skewed ecological predictors to reduce the influence of extreme outliers on model accuracy (Additional file 3: Table S1). To account for unmeasured spatial and temporal variation in mosquito presence, we also included collection latitude and longitude and the week, month, and year of each surveillance record as predictors. Furthermore, we included vector control agency and trap type as predictors to account for variation in surveillance methods.

### Model fitting

For each model, we randomly split observations into a training (80%) and testing (20%) dataset to evaluate model accuracy, where each observation was a surveillance record from a single collection date and location. Using the training data, we tuned model hyperparameters using fivefold cross validation and Bayesian optimization implemented with *rBayesianOptimization*. Specifically, we tuned the maximum tree depth, learning rate, and gamma (which controls regularization), as these parameters typically have the largest impact on model performance [80]. We provided initial hyperparameter values aimed at reducing overfitting (i.e. shallow trees, low learning rates, and high regularization). For cross validation, we used log loss as the model learning objective and ended training on the validation set after 10 rounds if no reductions in log loss were made. Additionally, we corrected for unbalanced classes (i.e. an unequal number of presence and absence records) in the less common species by setting the model parameter ‘scale_pos_weight’ equal to the square root of the total number of absence records divided by the total number of presence records for each species ([37]; Additional file 4: Table S2). We then fit an XGboost classification model using these optimal hyperparameters (Additional file 4: Table S2). As model output can vary based on the subsample of the data used, we conducted 100 iterations of the final model fitting, bootstrapping with a random 80% subset of the full dataset each time. This enabled us to generate confidence intervals for the model evaluation and predictor importance metrics (described below).

### Model evaluation and predictor importance

To evaluate the model performance, we used the withheld test data set and calculated AUC, which captures the model’s ability to accurately distinguish between classes (here, vector presence vs, absence), using *pROC*. To identify which predictors were most important for model performance, we estimated variable importance using XGboost. Specifically, we used ‘gain’—the relative improvement in model performance when adding a split in the tree on a given variable. We calculated these metrics for each of the 100 final model fits.

## Results

### Identifying key vector species

**Fig. 2:**
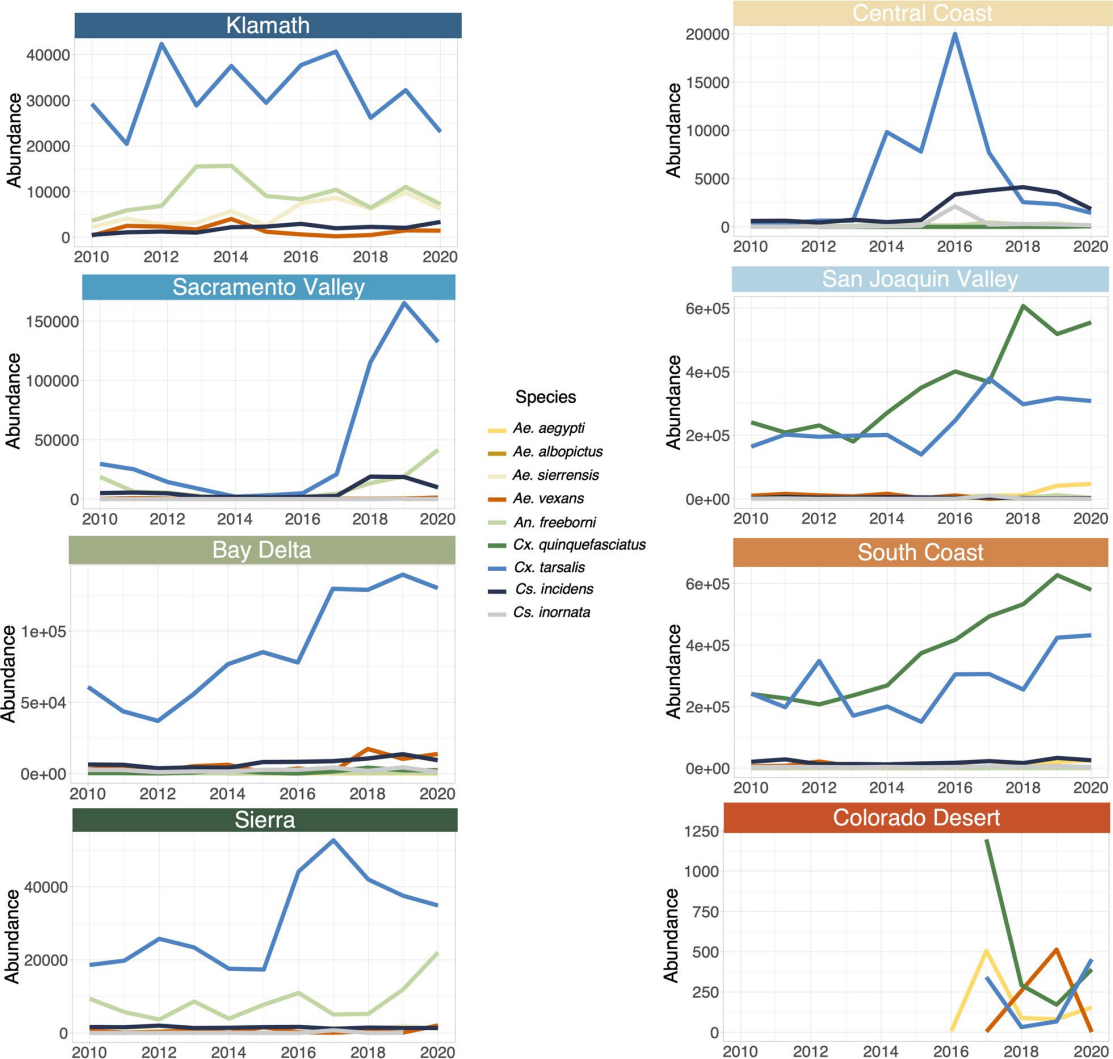
Annual trends in abundance of putative vector species for each California bioregion from 2012 to 2020. Abundance here denotes the total count of trapped adult female mosquitoes of a given species. Note: y-axes scales vary between bioregions

The vector species that were significantly, positively associated with dog heartworm cases varied regionally and included four species in total: *Ae. aegypti* (Central California; *p* = 0.010), *Ae. albopictus* (Southern California; *p* = 0.008), *Ae. sierrensis* (Central California; *p* = 0.045), and *Cs. incidens* (Northern and Central California; *p* = 0.005, 0.006) (Table 2). The effect sizes of these associations varied from an additional 8–35 cases in the year following a one standard deviation increase in mosquito abundance (Table 2). Of these key species, only *Cs. incidens* is consistently found at relatively high abundance in the given region, while the other identified vectors are relatively less common (Fig. 2, Additional file 2: Figure S2). Several mosquito species were significantly negatively associated with dog heartworm cases: *Culex tarsalis* in Northern California and *Ae. sierrensis* and *Ae. vexans* in Southern California (Table 2). Cases were also negatively associated with *Ae. albopictus* in Northern California, but this species was only found here in one bioregion and 1 year (Klamath in 2020) so this estimated relationship may be biased. The annual abundance of other putative vector species*—An. freeborni, Cs. inornata*, and *Cx. quinquefasciatus*—was not significantly associated with variation in dog heartworm cases in any region.

**Table 2:** Effect of mosquito predictors on dog heartworm cases

	Northern California			Central California			Southern California		
	Coef. estimate	Std. error	*p*-value	Coef. estimate	Std. error	*p*-value	Coef. estimate	Std. error	*p*-value
*Ae. aegypti*	−69.392	44.763	0.124	7.697	2.899	0.010*	26.229	13.939	0.070
*Ae. albopictus*	−1368.483	486.612	0.006*	NA	NA	NA	35.1330	12.444	0.008*
*Ae. sierrensis*	4.607	12.626	0.716	4.003	1.951	0.045*	−20.068	9.353	0.040*
*Ae. vexans*	12.912	17.754	0.469	3.258	1.869	0.087	−19.892	8.554	0.027*
*An. freeborni*	0.182	12.917	0.989	0.143	2.108	0.946	−8.314	8.373	0.329
*Cs. incidens*	34.238	11.822	0.005*	8.968	3.152	0.006*	−0.336	9.536	0.972
*Cs. inornata*	2.829	11.388	0.804	−0.027	2.007	0.989	−18.527	15.271	0.235
*Cx. quinquefasciatus*	−20.712	10.901	0.060	3.219	2.619	0.224	−30.629	15.727	0.061
*Cx. tarsalis*	−25.425	11.206	0.025*	−0.965	2.487	0.699	−4.221	16.298	0.797
*Dog density*	−0.874	8.092	0.914	−13.899	4.077	0.001*	62.701	20.786	0.005*
*Income*	−10.128	16.388	0.538	9.784	5.789	0.097	−81.849	16.851	< 0.001*
Full model	${R}^{2}$= 0.505			${R}^{2}$= 0.720			${R}^{2}$= 0.874		
Year and bioregion dummy variables only	${R}^{2}$= 0.409			${R}^{2}$= 0.499			${R}^{2}$= 0.598		
Year dummy variable only	${R}^{2}$= 0.405			${R}^{2}$= 0.492			${R}^{2}$= 0.503		
Bioregion dummy variable only	${R}^{2}$= 0.401			${R}^{2}$= 0.505			${R}^{2}$= 0.617		

Coefficient estimates, standard errors, and *p*-values for each predictor included in models of dog heartworm cases. Scaled coefficient estimates shown here denote the change in dog heartworm cases from a one standard deviation change in the predictor. Coefficients are scaled so that effects of different predictors are directly comparable. Statistically significant (*p* < 0.05) coefficients are denoted with *. Adjusted ${R}^{2}$ values are shown for four different model specifications: the full model including all predictors, a model including only the year and bioregion dummy variables, and a model including just the year, or just the bioregion dummy variable. *Aedes albopictus* was not included as a predictor in the model for Central California as it was not found in either Central California bioregion in any year.

Despite the associations described above, most of the variation in dog heartworm cases in all regions was explained by the bioregion and year dummy variables (together explaining 40.9%, 49.9%, and 59.8% of the variation in Northern, Central, and Southern California, respectively; Table 2), indicating that there is a large amount of unobserved heterogeneity at this level impacting cases. We found that estimated dog density had a significant, positive association with dog heartworm cases in Southern California, but a negative association in Central California (Table 2). However, changes in the density of other host species such as coyotes and feral dogs, which were not included in our model, may be driving additional bioregion- or year-level variation. Furthermore, median household income was only significantly associated with dog heartworm cases in Southern California, but other factors related to income such as the use of prophylactics or pet relinquishment rates, which were not included here, may also be contributing to the spatial and temporal variation.

### Ecological drivers of vector presence

#### Classification model accuracy

The classification models predicting the presence or absence of each vector species in a given trap location and date had high performance with a mean out-of-sample AUC > 0.93 for all species (Additional file 5: Table S3). The classification model sensitivity—the ability to accurately predict vector presence—varied from 0.674 to 0.993 and was highest for the most abundant species, *Cx. quinquefasciatus* and *Cx. tarsalis*. Model specificity—the ability to accurately predict vector absence—was typically higher, ranging from 0.686 to 0.995, and was highest for the less abundant species, all *Aedes* spp., *An. freeborni*, and *Cs. inornata* (Additional file 5: Table S3).

#### Predictor importance

**Fig. 3:**
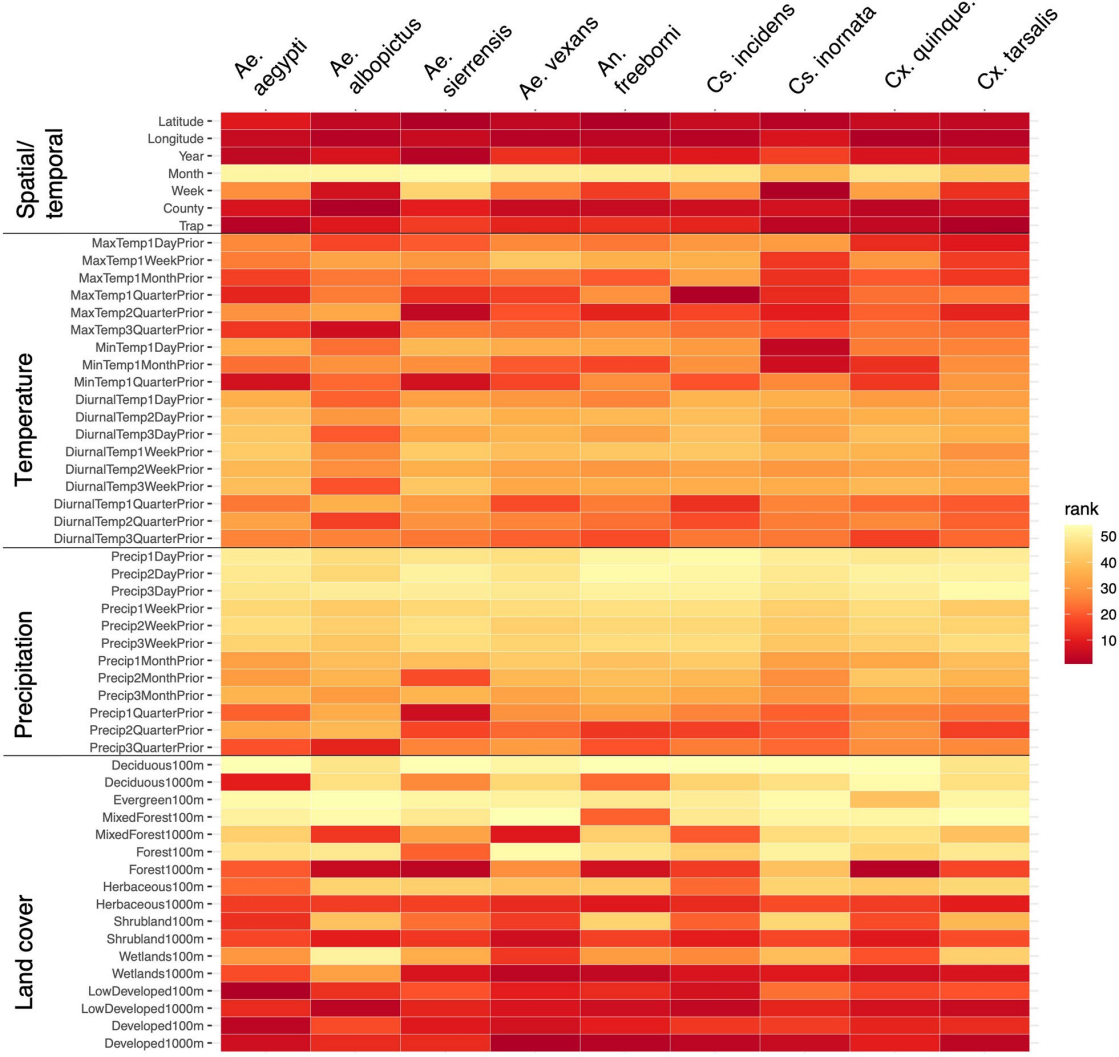
Variable importance in predicting vector species presence/absence. For each species, predictors are ranked based on their mean gain across the 100 model iterations

For the four species identified as positively associated with dog heartworm cases in at least one region (*Ae. aegypti, Ae. albopictus, Ae. sierrensis*, and *Cs. incidens*), the proportions of developed land cover in the region near the trap, as well as maximum and minimum temperature in the preceding seasons, were consistently among the top 10 predictors associated with their presence or absence (Fig. 3, Table 3). For *Ae. aegypti*, presence was associated with a greater proportion of developed cover (of both low and high intensity) in the region surrounding the trap as well higher minimum daily temperatures in the preceding winter. Conversely, *Ae. sierrensis* presence was associated with less developed and more forested land cover as well as cooler, wetter conditions in the preceding winter. For *Ae. albopictus* and *Cs. incidens,* we found a likely association with neighborhoods and human dwellings in less urbanized settings: presence was associated with higher proportions of low-intensity developed cover (e.g. areas with a mixture of constructed materials and vegetation, most commonly single-family neighborhoods) in the immediate surroundings (100 m), but less developed cover (of both low and high intensity) in the surrounding kilometer. Furthermore, *Cs. incidens* presence was associated with lower maximum daily temperatures in the preceding winter, while *Ae. albopictus* presence was associated with higher maximum temperatures in the preceding summer. In general, the proportions of low and high intensity developed land cover surrounding the trap were frequently included among the most important in predicting presence or absence for the nine putative vector species, while diurnal temperature range was not an important predictor for any species.

**Table 3:** Mean values of ecological predictors for vector presence and absence

	*Ae* *aegypti*		*Ae. albopictus*		*Ae. sierrensis*		*Ae* *vexans*		*An. freeborni*		*Cs. incidens*		*Cs* *inornata*		*Cx* *quinquefasciatus*		*Cx* *tarsalis*	
Absence/ presence	0	1	0	1	0	1	0	1	0	1	0	1	0	1	0	1	0	1
Max temp 1 day prior	28.93	32.22	29.21	29.31	29.25	28.12	29.15	31.37	29.08	30.92	29.99	26.52	29.46	24.33	28.63	29.61	28.17 *	30.19*
Max temp 1 quarter prior	28.74	32.25	29.03	29.35	27.18	22.02	28.98	31.12	28.92	30.56	29.83*	26.27*	29.29	24.18	28.44	29.44	28.08	29.93
Max temp 2 quarters prior	28.45	32.27	28.76	29.39	21.52	15.25	28.70	31.05	28.66	30.17	29.62	25.81	29.00*	24.28 *	28.12	29.21	27.92	29.56
Max temp 3 quarters prior	28.21	32.33	28.55*	29.24*	21.87	21.99	28.49	31.01	28.45	29.96	29.48	25.36	28.76	24.53	27.89	29.01	27.77	29.29
Min temp 1 day prior	28.29	32.28	28.62	29.26	14.37	11.33	28.56	31.03	28.52	30.02	29.52	25.53	28.84*	24.50*	27.96	29.08	27.81	29.39
Min temp 1 month prior	26.80	31.56	27.18	28.41	14.12	10.07	27.11	30.25	27.13	28.03	28.23	23.62	27.28*	25.43*	26.34	27.78	26.73	27.62
Min temp 1 quarter prior	24.72*	29.27*	25.10	25.93	12.98*	7.86 *	25.02	28.34	25.10	25.10	26.10	21.66	25.06	25.88	24.09	25.81	25.04	25.16
Precip 1 quarter prior	13.69	16.95	13.94	15.82	2.86 *	4.79 *	13.94	14.44	14.02	13.05	14.38	12.48	14.14	10.37	12.71	14.83	13.95	13.96
Deciduous 1000 m	14.56*	15.32*	14.63	13.41	0.00	0.01	14.57	16.52	14.46	16.91	15.10	12.98	14.65	14.06	15.20	14.21	13.84	15.36
Mixed forest 1000 m	14.60	15.33	14.67	13.44	0.00	0.03	14.61*	16.59*	14.50	16.97	15.13	13.05	14.69	14.12	15.25	14.25	13.86	15.42
Forest 100 m	1.34	0.57	1.28	0.70	0.00	0.08	1.28	1.39	1.28	1.25	1.18	1.63	1.24	2.07	1.38	1.20	1.38	1.18
Forest 1000 m	14.80	15.50	14.87*	13.70*	0.01 *	0.11 *	14.81	16.77	14.69*	17.24*	15.32	13.28	14.89	14.29	15.50*	14.42*	14.01	15.67
Herbaceous 1000 m	12.79	12.90	12.81	11.36	0.04	0.15	12.76	14.32	12.78*	13.13*	13.11	11.74	12.76	13.66	12.69	12.88	12.60*	12.99*
Shrubland 1000 m	14.14	14.86	14.21*	12.92*	0.04	0.12	14.15*	16.10*	14.06	16.14	14.66*	12.62*	14.22	13.90	14.64*	13.90*	13.52	14.84
Wetlands 100 m	136.28	93.27	132.65	147.65	0.03	0.06	133.08	119.59	127.13	212.03	118.07	182.75	135.23	85.65	187.50	94.41	113.87	150.73
Wetlands 1000 m	14.65	15.37	14.71	13.53	0.02 *	0.06 *	14.66*	16.65*	14.54*	17.01*	15.17*	13.12*	14.73*	14.16*	15.31*	14.29*	13.91*	15.46*
Low developed 100 m	121.57 *	125.71 *	121.37	213.71	0.32	0.32	121.97 *	119.69 *	115.83	207.84	120.14 *	127.95 *	123.64	89.30	168.70	89.17	105.46	137.59
Low developed 1000 m	14.16	14.93	14.23 *	13.01*	0.23	0.22	14.17*	16.15*	14.08*	16.24*	14.70*	12.60*	14.24	13.87	14.68*	13.91*	13.53*	14.88*
Developed 100 m	14.91*	15.56*	14.97	13.78	0.40*	0.10*	14.91*	17.02*	14.78*	17.51*	15.42	13.39	14.99	14.53	15.62	14.51	14.07	15.81
Developed 1000 m	13.66*	14.32*	13.72	12.32	0.41	0.14	13.66*	15.55*	13.61*	15.19*	14.15*	12.20*	13.71*	13.70*	13.97*	13.53*	13.17*	14.22*

Mean values of ecological predictors for when a given vector species are absent (0) or present (1). Predictors that were ranked as among the top 10 most important, based on mean gain, for that species are denoted with a*. Only predictors included in the top 10 most important for any species are included here. See Additional file 6: Table S4 for predictor rankings and Additional file 7: Table S5 for the full list of ecological predictors

## Discussion

Identifying the ecological drivers of dog heartworm transmission is critical for disease prevention. Vector presence, climate, and land cover conditions are known to be key drivers, but the specific species and abiotic factors influencing transmission in a particular setting are often unknown and challenging to identify [10, 25]. Here, we leverage a comprehensive dog heartworm case data set as well as long-term, finely resolved vector surveillance data to investigate the vector species, climate, and land cover features associated with dog heartworm transmission in California. Using a statistical modeling approach that controls for unobserved spatial and temporal variation in cases, we identify four vectors of dog heartworm transmission in different regions of California: *Culiseta incidens* in Northern and Central California, *Aedes aegypti* and *Ae. sierrensis* in Central California only, and *Aedes albopictus* in Southern California only. Using flexible machine learning models, we find that the proportions of developed cover, and minimum and maximum daily temperature in preceding seasons, are the strongest drivers of these species’ presence or absence.

Over 25 mosquito species have been identified as potential vectors of dog heartworm in the US, including nine species in California [11]. While *Aedes sierrensis* is often described as the state’s principal vector [59, 68, 81] because of its widespread distribution, we found its abundance was only significantly associated with dog heartworm cases in Central California. We found that, despite being broadly distributed, *Ae. sierrensis* is not present at high abundance in any bioregion. Furthermore, prior studies have found low infection prevalence in field-collected *Ae. sierrensis* adults in California [56, 65] and low rates of dog-biting in some regions [60]. Thus, discrepancies in the relative importance of *Ae. sierrensis* in dog heartworm transmission could be due to the type of evidence being considered (e.g. physiological competence, abundance, distribution, infection prevalence, host-biting rates) and/or regional variation in these factors.

*Ae. aegypti*, *Ae. albopictus*, and *Cs. incidens* have not been previously been considered major vectors of dog heartworm in California. For each of these species, their physiological vector competence (e.g. ability to acquire and maintain *D. immitis*) has been established by prior laboratory studies in other regions [39, 41, 42, 46, 48–51, 57, 71, 82]. However, their rates of vector efficiency have varied based on the mosquito strain tested [49, 51, 57] and were generally low in the case of *Ae. aegypti* [38, 39, 41, 42]. For *Cs. incidens,* natural infection with *D. immitis* [65, 68] and a willingness to bite dogs [57, 61, 65] have been observed in California populations specifically. Furthermore, the species is widely distributed across the state and has been previously identified as a potentially important secondary vector of dog heartworm [57, 65, 71]. Our finding that *Cs. incidens* abundance is significantly positively associated with dog heartworm cases in Northern and Central California suggests that this species may play an under-recognized role in transmission in these regions. For *Ae. albopictus*, prior studies in the Eastern and Midwestern US found moderate rates of infection in field-collected adults [16, 17, 46, 47] and high rates of dog-biting [52, 53]. Similarly, natural infection and high rates of dog-biting have been observed in *Ae. aegypti* in the Southern US [43, 45] as well as Mexico and Argentina [44, 83]. *Aedes albopictus* and *Ae. aegypti* were rarely detected in California prior to their establishment in 2011 and 2015, respectively [84]. Since then, both species have rapidly increased in abundance and have become established in Coastal, Central, and Southern California [85]. However, to our knowledge, no studies have yet evaluated the vector competence, field infection rates, or dog-biting rates of these species in California. Our finding that *Ae. aegypti* and *Ae. albopictus* are significantly positively associated with dog heartworm cases in Central and Southern California, respectively, highlights their potential role in dog heartworm transmission in these regions and the need for further investigation of these species’ vector potential.

While our analysis of the vector species associated with dog heartworm included nearly a decade of mosquito surveillance and case data, we cannot definitively claim that *Ae. aegypti, Ae. albopictus, Ae. sierrensis*, and *Cs. incidens* are the key species driving transmission in California. In particular, the available heartworm case data include only antigen test results from domestic animals, thus not capturing heartworm transmission intensity in wild animals. Furthermore, as cases are assigned to the county from which the test was reported, local and imported cases cannot be distinguished. Our statistical modeling approach, which examined differences in cases over time for a particular bioregion and controlled for annual variation in cases, could address some of these limitations. However, an ideal investigation would incorporate information on field infection prevalences and host-biting rates in addition to vector abundances and distributions. This more rigorous investigation is hampered by the lack of systematic parasite surveillance in mosquitoes in California, and the US as a whole, likely due to the high cost of testing many different species and individuals. However, California has a uniquely comprehensive vector surveillance system (CalSurv), which includes finely resolved spatial and temporal data on mosquito abundances across much of the state, dating back decades for many locations and species. Leveraging this rich data source, we provide a critical first step towards understanding the vectors involved in dog heartworm transmission in the Western US, a region that has broadly experienced an increase in dog heartworm cases in the past decade [29].

In investigating the ecological drivers of vector presence, we found that land cover feature, namely the proportion of developed land cover in the region surrounding the surveillance site, was consistently one of the strongest predictors of a given vector species’ presence. Of the four species associated with higher heartworm prevalence, we found *Ae. aegypti* was more common in highly developed areas (e.g. apartment complexes or commercial settings), while *Ae. sierrensis* was more common in less developed, more forested areas—findings that match prior habitat associations of these two species [86–89]. *Culiseta incidens* and *Ae. albopictus* were both more likely in areas with some human development in the immediate surroundings, but with less high intensity development in the broader environment. This is also consistent with prior findings that these species are typically found in peridomestic settings [62, 88, 90]. Given these species-specific land cover associations, strategies for targeted vector surveillance may vary by region (e.g. prioritizing placing traps directly within areas of low intensity development in Northern and Southern California to target *Cs. incidens* and *Ae. albopictus*, respectively), while efforts to survey a wider range of potential vectors would aim to place traps in a mixture of land cover types.

## Conclusions

As cases of heartworm disease are increasing across much of the US, including California, a better understanding of the drivers of transmission is needed to protect companion and wild animals. Mosquito vector presence has been identified as the main risk factor for transmission, but identifying the primary vector species in a specific region is challenging given the large number of putative vector species and spatial variation in their ecologies and vector status. Here, we leveraged long-term mosquito surveillance, climate, and land cover data to identify four regionally specific vector species—*Ae. aegypti* (Central California), *Ae. albopictus* (Southern California), *Ae. sierrensis* (Central California), and *Cs. incidens* (Northern and Central California)—and their local habitat associations. Investigating natural infection prevalence and host biting rates in these species, as well as other local vectors, is an important next step in understanding the local transmission ecology. Doing so will enable more targeted and effective vector control and disease prevention.

## Supplementary Information

**Additional file 1: Figure S1. **Correlation matrix indicating correlations between the annual, county-level abundance of different mosquito species.

**Additional file 2: Figure S2. **Relative abundance of each mosquito species by bioregion. Abundance here reflects the average number of trapped adult females of a given species between 2010–2020.

**Additional file 3: Table S1. **Ecological predictors retained for analysis (n = 47). Predictors listed in the Methods that are not included in this list were removed because of high collinearity (pairwise correlations > 0.90 with an included predictor). These ecological predictors were included in models along with the year, month, week, latitude, and longitude of surveillance to account for spatiotemporal variation and trap type and vector control agency to account for variation in surveillance methods.

**Additional file 4: Table S2. **Optimal hyperparameters for models predicting the presence/absence of each vector species, identified using Bayesian optimization.

**Additional file 5: Table S3. **Performance metrics for models predicting species presence/absence in a given trap night and location. Values shown here for AUC, sensitivity, specificity, accuracy, and balanced accuracy are the mean values from the 100 model iterations. Values are shown for the full model, which contains all the ecological and spatiotemporal predictors for a given species, as well as for null models predicting all ‘present’ or all ‘absent.’

**Additional file 6: Table S4. **Variable importance for each predictor in models predicting the presence/absence of each vector species. The left column under each species indicates the mean gain from the 100 model iterations. The right column indicates the predictor rank (i.e. 1–54) based on mean gain.

**Additional file 7: Table S5. **Mean values of the ecological predictors for when a given vector species is absent (0) or present (1). Predictors that were ranked as among the top 10 most important, based on mean gain, for that species are denoted with a *.

## Data Availability

All data and code used in this study are available on GitHub in the following repository: https://github.com/lcouper/DogHeartworm.
