# Supplementary material for: Ecological drivers of dog heartworm transmission in California
Source: Parasit Vectors. 2022 Oct 23;15:388. doi: 10.1186/s13071-022-05526-x (PMC9590206; doi:10.1186/s13071-022-05526-x)
Supplement: Supplementary file 1 — Additional file 1: Figure S1. Correlation matrix indicating correlations between the annual, county-level abundance of different mosquito species. [file 13071_2022_5526_MOESM1_ESM.docx]

**Supplementary Material**

**Additional File 1**

**
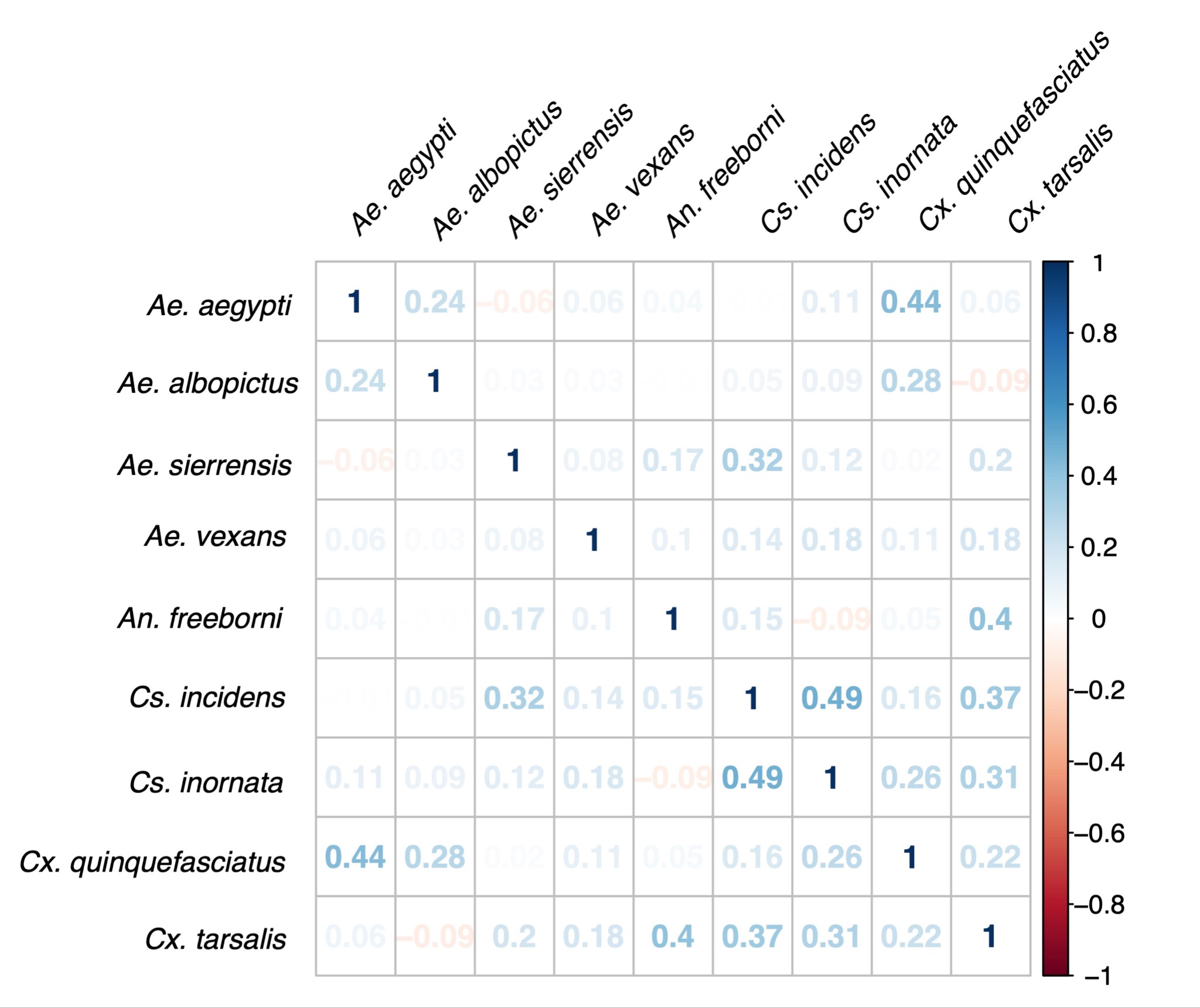
**

**Figure S1.** Correlation matrix indicating correlations between the annual, county-level abundance of different mosquito species.
