# Supplementary material for: Ecological drivers of dog heartworm transmission in California
Source: Parasit Vectors. 2022 Oct 23;15:388. doi: 10.1186/s13071-022-05526-x (PMC9590206; doi:10.1186/s13071-022-05526-x)
Supplement: Supplementary file 2 — Additional file 2: Figure S2. Relative abundance of each mosquito species by bioregion. Abundance here reflects the average number of trapped adult females of a given species between 2010–2020. [file 13071_2022_5526_MOESM2_ESM.docx]

**Additional File 2**

**
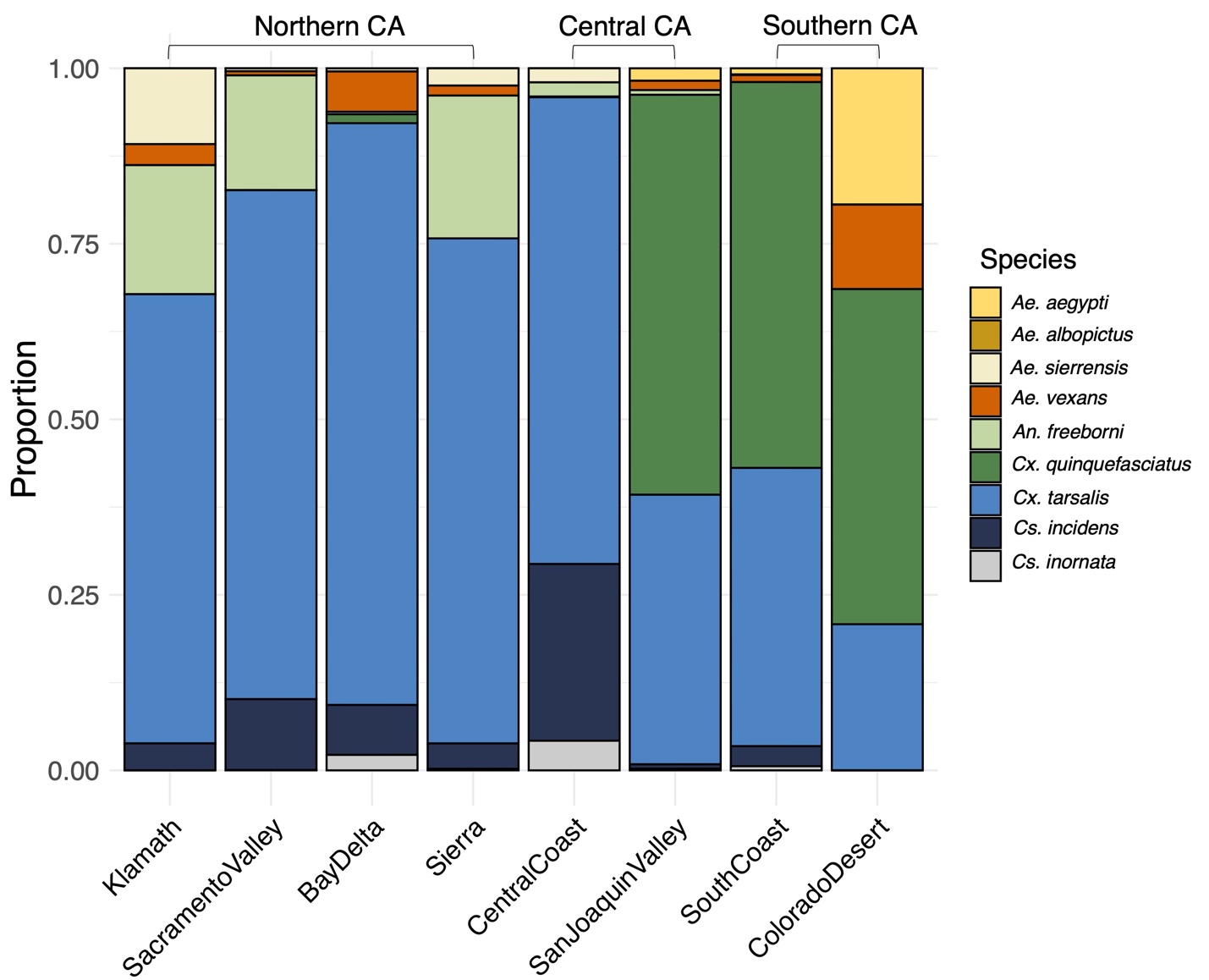
**

**Figure S2.** Relative abundance of each mosquito species by bioregion. Abundance here reflects the average number of trapped adult females of a given species between 2010 - 2020.
