# Supplementary material for: Ecological drivers of dog heartworm transmission in California
Source: Parasit Vectors. 2022 Oct 23;15:388. doi: 10.1186/s13071-022-05526-x (PMC9590206; doi:10.1186/s13071-022-05526-x)
Supplement: Supplementary file 3 — Additional file 3: Table S1. Ecological predictors retained for analysis (n = 47). Predictors listed in the Methods that are not included in this list were removed because of high collinearity (pairwise correlations > 0.90 with an included predictor). These ecological predictors were included in models along with the year, month, week, latitude, and longitude of surveillance to account for spatiotemporal variation and trap type and vector control agency to account for variation in surveillance methods. [file 13071_2022_5526_MOESM3_ESM.docx]

**Additional File 3**

**Table S1.** Ecological predictors retained for analysis (n = 47). Predictors listed in the Methods that are not included in this list were removed due to high collinearity (pairwise correlations >0.90 with an included predictor). These ecological predictors were included in models along with the year, month, week, latitude, and longitude of surveillance to account for spatiotemporal variation, and trap type and vector control agency to account for variation in surveillance methods.

| **Temperature** | **Precipitation** | **Land cover (proportion cover)** |
| --- | --- | --- |
| Max temp 1 day prior | Precip 1 day prior (log) | Forest 100 m (log) |
| Max temp 1 week prior | Precip 2 days prior (log) | Forest 1000 m (log) |
| Max temp 1 month prior | Precip 3 days prior (log) | Deciduous 100 m (log) |
| Max temp 1 quarter prior | Precip 1 week prior (log) | Deciduous 1000 m (log) |
| Max temp 2 quarters prior | Precip 2 weeks prior (log) | Evergreen 100 m (log) |
| Max temp 3 quarters prior | Precip 3 weeks prior (log) | Mixed forest 100 m (log) |
| Min temp 1 day prior | Precip 1 month prior (log) | Mixed forest 1000 m (log) |
| Min temp 1 month prior | Precip 2 months prior (log) | Herbaceous 100 m (log) |
| Min temp 1 quarter prior | Precip 3 months prior (log) | Herbaceous 1000 m (log) |
| Diurnal temp 1 day prior | Precip 1 quarter prior (log) | Shrubland 100 m (log) |
| Diurnal temp 2 days prior | Precip 2 quarters prior | Shrubland 1000 m (log) |
| Diurnal temp 3 days prior | Precip 3 quarters prior | Wetlands 100 m (log) |
| Diurnal temp 1 week prior |  | Wetlands 1000 m (log) |
| Diurnal temp 2 weeks prior |  | Low developed 100 m (log) |
| Diurnal temp 3 weeks prior |  | Low developed 1000 m (log) |
| Diurnal temp 1 quarter prior |  | Developed 100 m (log) |
| Diurnal temp 2 quarters prior |  | Developed 1000 m (log) |
| Diurnal temp 3 quarters prior |  |  |
