# Supplementary material for: Ecological drivers of dog heartworm transmission in California
Source: Parasit Vectors. 2022 Oct 23;15:388. doi: 10.1186/s13071-022-05526-x (PMC9590206; doi:10.1186/s13071-022-05526-x)
Supplement: Supplementary file 4 — Additional file 4: Table S2. Optimal hyperparameters for models predicting the presence/absence of each vector species, identified using Bayesian optimization. [file 13071_2022_5526_MOESM4_ESM.docx]

**Additional File 4**

**Table S2.** Optimal hyperparameters for models predicting the presence/absence of each vector species, identified using Bayesian optimization

| **Species** | **Learning rate (eta)** | **Maximum tree depth** | **Regularization (gamma)** | **Class weights (scale_pos_weight)** |
| --- | --- | --- | --- | --- |
| *Ae. aegypti* | 0.30 | 10.00 | 10.00 | 3.34 |
| *Ae. albopictus* | 0.18 | 10.00 | 10.89 | 13.06 |
| *Ae. sierrensis* | 0.30 | 10.00 | 10.00 | 4.88 |
| *Ae. vexans* | 0.30 | 10.00 | 10.00 | 6.27 |
| *An. freeborni* | 0.23 | 10.00 | 10.00 | 3.76 |
| *Cs. incidens* | 0.30 | 10.00 | 10.00 | 1.85 |
| *Cs. inornata* | 0.19 | 10.00 | 13.53 | 4.35 |
| *Cx. quinquefasciatus* | 0.27 | 10.00 | 12.27 | 1.0 |
| *Cx. tarsalis* | 0.30 | 10.00 | 10.00 | 1.0 |
