# Supplementary material for: Ecological drivers of dog heartworm transmission in California
Source: Parasit Vectors. 2022 Oct 23;15:388. doi: 10.1186/s13071-022-05526-x (PMC9590206; doi:10.1186/s13071-022-05526-x)
Supplement: Supplementary file 5 — Additional file 5: Table S3. Performance metrics for models predicting species presence/absence in a given trap night and location. Values shown here for AUC, sensitivity, specificity, accuracy, and balanced accuracy are the mean values from the 100 model iterations. Values are shown for the full model, which contains all the ecological and spatiotemporal predictors for a given species, as well as for null models predicting all ‘present’ or all ‘absent.’ [file 13071_2022_5526_MOESM5_ESM.docx]

**Additional File 5**

**Table S3.** Performance metrics for models predicting species presence/absence in a given trap night and location. Values shown here for AUC, sensitivity, specificity, accuracy, and balanced accuracy are the mean values from the 100 model iterations. Values are shown for the full model, which contains all the ecological and spatiotemporal predictors for a given species, as well as for null models predicting all ‘present’ or all ‘absent.’

|  | *Ae. aegypti* | *Ae. albopictus* | *Ae. sierrensis* | *Ae. vexans* | *An. freeborni* | *Cs. incidens* | *Cs. inornata* | *Cx. quinquefasciatus* | *Cx. tarsalis* |
| --- | --- | --- | --- | --- | --- | --- | --- | --- | --- |
| Full model | | | | | | | | | |
| AUC | 0.986696 | 0.996501 | 0.986803 | 0.97994 | 0.974906 | 0.942162 | 0.952195 | 0.985649 | 0.930227 |
| Sensitivity | 0.891433 | 0.821666 | 0.826579 | 0.754370 | 0.796321 | 0.808078 | 0.674111 | 0.992916 | 0.938034 |
| Specificity | 0.966442 | 0.995400 | 0.983077 | 0.986386 | 0.963654 | 0.906978 | 0.964410 | 0.853333 | 0.686191 |
| Accuracy | 0.960268 | 0.994385 | 0.976775 | 0.980555 | 0.952588 | 0.884511 | 0.949825 | 0.935432 | 0.815111 |
| Balanced Accuracy | 0.928938 | 0.908533 | 0.904828 | 0.870378 | 0.879983 | 0.857528 | 0.819261 | 0.923125 | 0.812113 |
| Null model - all presence | | | | | | | | | |
| AUC | 0.50 | 0.50 | 0.50 | 0.50 | 0.50 | 0.50 | 0.50 | 0.50 | 0.50 |
| Sensitivity | 1.00 | 1.00 | 1.00 | 1.00 | 1.00 | 1.00 | 1.00 | 1.00 | 1.00 |
| Specificity | 0.00 | 0.00 | 0.00 | 0.00 | 0.00 | 0.00 | 0.00 | 0.00 | 0.00 |
| Accuracy | 0.0823 | 0.0058 | 0.0403 | 0.0251 | 0.0661 | 0.2267 | 0.0502 | 0.5883 | 0.5119 |
| Balanced Accuracy | 0.50 | 0.50 | 0.50 | 0.50 | 0.50 | 0.50 | 0.50 | 0.50 | 0.50 |
| Null model – all absence | | | | | | | | | |
| AUC | 0.50 | 0.50 | 0.50 | 0.50 | 0.50 | 0.50 | 0.50 | 0.50 | 0.50 |
| Sensitivity | 0.00 | 1.00 | 0.00 | 1.00 | 1.00 | 1.00 | 1.00 | 1.00 | 1.00 |
| Specificity | 1.00 | 0.00 | 1.00 | 0.00 | 0.00 | 0.00 | 0.00 | 0.00 | 0.00 |
| Accuracy | 0.918 | 0.9942 | 0.9597 | 0.9749 | 0.9339 | 0.7733 | 0.9498 | 0.4117 | 0.4881 |
| Balanced Accuracy | 0.50 | 0.50 | 0.50 | 0.50 | 0.50 | 0.50 | 0.50 | 0.50 | 0.50 |
