# Supplementary material for: Ecological drivers of dog heartworm transmission in California
Source: Parasit Vectors. 2022 Oct 23;15:388. doi: 10.1186/s13071-022-05526-x (PMC9590206; doi:10.1186/s13071-022-05526-x)
Supplement: Supplementary file 6 — Additional file 6: Table S4. Variable importance for each predictor in models predicting the presence/absence of each vector species. The left column under each species indicates the mean gain from the 100 model iterations. The right column indicates the predictor rank (i.e. 1–54) based on mean gain. [file 13071_2022_5526_MOESM6_ESM.docx]

**Additional File 6**

**Table S4.** Variable importance for each predictor in models predicting the presence/absence of each vector species. The left column under each species indicates the mean gain from the 100 model iterations. The right column indicates the predictor rank (*i.e.,* 1-54) based on mean gain.

|  | *Ae. aegypti* | | *Ae. albopictus* | | *Ae. sierrensis* | | *Ae.*  *vexans* | | *An.*  *freeborni* | | *Cs. incidens* | | *Cs. inornata* | | *Cx.*  *quinquefasciatus* | | *Cx.*  *tarsalis* | |
| --- | --- | --- | --- | --- | --- | --- | --- | --- | --- | --- | --- | --- | --- | --- | --- | --- | --- | --- |
| Latitude | 0.0185 | 9 | 0.0169 | 4 | 0.4390 | 1 | 0.0583 | 4 | 0.4474 | 1 | 0.0782 | 5 | 0.1190 | 2 | 0.0374 | 5 | 0.0741 | 4 |
| Longitude | 0.0355 | 5 | 0.0343 | 2 | 0.0376 | 5 | 0.1131 | 2 | 0.0799 | 3 | 0.1554 | 2 | 0.0335 | 8 | 0.7194 | 1 | 0.1252 | 2 |
| Year | 0.0609 | 4 | 0.0092 | 8 | 0.0837 | 2 | 0.0154 | 13 | 0.0223 | 8 | 0.0271 | 9 | 0.0122 | 16 | 0.0072 | 8 | 0.0328 | 7 |
| Month | 0.0001 | 52 | 0.0001 | 52 | 0.0003 | 53 | 0.0008 | 50 | 0.0005 | 50 | 0.0009 | 48 | 0.0050 | 37 | 0.0003 | 48 | 0.0026 | 41 |
| Week | 0.0013 | 28 | 0.0103 | 7 | 0.0028 | 44 | 0.0079 | 25 | 0.0083 | 15 | 0.0041 | 28 | 0.1257 | 1 | 0.0013 | 32 | 0.0147 | 13 |
| County | 0.0219 | 8 | 0.7452 | 1 | 0.0176 | 10 | 0.0480 | 5 | 0.0326 | 5 | 0.0763 | 6 | 0.0345 | 7 | 0.0402 | 3 | 0.0560 | 6 |
| Trap | 0.1998 | 2 | 0.0092 | 9 | 0.0108 | 15 | 0.0200 | 11 | 0.0127 | 13 | 0.0233 | 11 | 0.1097 | 3 | 0.0388 | 4 | 0.2045 | 1 |
| Max temp 1 day prior | 0.0014 | 27 | 0.0048 | 17 | 0.0060 | 20 | 0.0071 | 27 | 0.0055 | 24 | 0.0034 | 30 | 0.0059 | 31 | 0.0036 | 12 | 0.0186 | 9 |
| Max temp 1 month prior | 0.0037 | 16 | 0.0031 | 24 | 0.0058 | 22 | 0.0080 | 24 | 0.0061 | 20 | 0.0032 | 32 | 0.0169 | 13 | 0.0025 | 20 | 0.0143 | 14 |
| Max temp 1 quarter prior | 0.0095 | 11 | 0.0030 | 25 | 0.0139 | 13 | 0.0132 | 16 | 0.0046 | 29 | 0.1642 | 1 | 0.0169 | 12 | 0.0022 | 23 | 0.0075 | 25 |
| Max temp 1 week prior | 0.0015 | 25 | 0.0025 | 33 | 0.0042 | 30 | 0.0044 | 41 | 0.0042 | 36 | 0.0027 | 36 | 0.0142 | 14 | 0.0014 | 30 | 0.0133 | 15 |
| Max temp 2 quarters prior | 0.0011 | 29 | 0.0023 | 34 | 0.0378 | 4 | 0.0092 | 19 | 0.0146 | 11 | 0.0119 | 17 | 0.0241 | 10 | 0.0023 | 21 | 0.0153 | 11 |
| Max temp 3 quarters prior | 0.0049 | 14 | 0.0127 | 6 | 0.0050 | 25 | 0.0081 | 23 | 0.0051 | 27 | 0.0050 | 23 | 0.0105 | 19 | 0.0019 | 24 | 0.0081 | 23 |
| Min temp 1 day prior | 0.0008 | 35 | 0.0031 | 23 | 0.0037 | 38 | 0.0052 | 35 | 0.0043 | 34 | 0.0034 | 31 | 0.0905 | 4 | 0.0018 | 25 | 0.0074 | 26 |
| Min temp 1 month prior | 0.0016 | 23 | 0.0028 | 29 | 0.0045 | 28 | 0.0091 | 20 | 0.0062 | 17 | 0.0037 | 29 | 0.0639 | 6 | 0.0034 | 13 | 0.0066 | 28 |
| Min temp 1 quarter prior | 0.0300 | 7 | 0.0032 | 22 | 0.0342 | 7 | 0.0128 | 17 | 0.0048 | 28 | 0.0064 | 19 | 0.0073 | 27 | 0.0034 | 14 | 0.0061 | 30 |
| Diurnal temp 1 day prior | 0.0007 | 36 | 0.0033 | 21 | 0.0042 | 32 | 0.0061 | 30 | 0.0053 | 26 | 0.0027 | 37 | 0.0054 | 36 | 0.0013 | 31 | 0.0058 | 32 |
| Diurnal temp 1 quarter prior | 0.0016 | 24 | 0.0021 | 36 | 0.0042 | 31 | 0.0118 | 18 | 0.0053 | 25 | 0.0153 | 13 | 0.0075 | 26 | 0.0023 | 22 | 0.0105 | 20 |
| Diurnal temp 1 week prior | 0.0005 | 42 | 0.0030 | 27 | 0.0030 | 42 | 0.0045 | 39 | 0.0032 | 41 | 0.0024 | 41 | 0.0048 | 38 | 0.0012 | 37 | 0.0062 | 29 |
| Diurnal temp 2 days prior | 0.0005 | 40 | 0.0028 | 30 | 0.0033 | 40 | 0.0051 | 36 | 0.0040 | 38 | 0.0027 | 39 | 0.0055 | 34 | 0.0012 | 36 | 0.0054 | 35 |
| Diurnal temp 2 quarters prior | 0.0009 | 33 | 0.0049 | 16 | 0.0045 | 29 | 0.0073 | 26 | 0.0057 | 23 | 0.0081 | 18 | 0.0080 | 25 | 0.0017 | 27 | 0.0088 | 21 |
| Diurnal temp 2 weeks prior | 0.0006 | 38 | 0.0029 | 28 | 0.0037 | 36 | 0.0060 | 32 | 0.0046 | 30 | 0.0031 | 33 | 0.0059 | 30 | 0.0013 | 33 | 0.0058 | 33 |
| Diurnal temp 3 days prior | 0.0005 | 41 | 0.0038 | 20 | 0.0038 | 34 | 0.0048 | 37 | 0.0043 | 33 | 0.0026 | 40 | 0.0058 | 33 | 0.0012 | 39 | 0.0053 | 36 |
| Diurnal temp 3 quarters prior | 0.0015 | 26 | 0.0030 | 26 | 0.0052 | 24 | 0.0086 | 21 | 0.0062 | 18 | 0.0047 | 24 | 0.0085 | 24 | 0.0033 | 16 | 0.0083 | 22 |
| Diurnal temp 3 weeks prior | 0.0005 | 39 | 0.0039 | 19 | 0.0032 | 41 | 0.0059 | 34 | 0.0043 | 35 | 0.0028 | 35 | 0.0054 | 35 | 0.0012 | 38 | 0.0056 | 34 |
| Precip 1 day prior | 0.0001 | 50 | 0.0004 | 46 | 0.0007 | 48 | 0.0009 | 47 | 0.0005 | 52 | 0.0004 | 53 | 0.0011 | 50 | 0.0003 | 49 | 0.0010 | 50 |
| Precip 1 month prior | 0.0010 | 49 | 0.0014 | 45 | 0.0037 | 51 | 0.0039 | 48 | 0.0034 | 53 | 0.0022 | 52 | 0.0058 | 49 | 0.0012 | 51 | 0.0042 | 51 |
| Precip 1 quarter prior | 0.0019 | 48 | 0.0022 | 50 | 0.0361 | 50 | 0.0065 | 49 | 0.0043 | 51 | 0.0045 | 51 | 0.0099 | 48 | 0.0018 | 50 | 0.0078 | 53 |
| Precip 1 week prior | 0.0002 | 45 | 0.0008 | 42 | 0.0017 | 45 | 0.0017 | 46 | 0.0012 | 47 | 0.0010 | 47 | 0.0027 | 43 | 0.0005 | 46 | 0.0025 | 42 |
| Precip 2 days prior | 0.0001 | 46 | 0.0004 | 43 | 0.0005 | 47 | 0.0009 | 43 | 0.0004 | 45 | 0.0004 | 45 | 0.0012 | 42 | 0.0002 | 45 | 0.0009 | 44 |
| Precip 2 months prior | 0.0010 | 44 | 0.0021 | 41 | 0.0066 | 46 | 0.0046 | 44 | 0.0036 | 46 | 0.0027 | 46 | 0.0063 | 41 | 0.0011 | 43 | 0.0052 | 46 |
| Precip 2 quarters prior | 0.0009 | 32 | 0.0020 | 39 | 0.0070 | 39 | 0.0082 | 42 | 0.0092 | 40 | 0.0135 | 42 | 0.0102 | 32 | 0.0017 | 34 | 0.0132 | 39 |
| Precip 2 weeks prior | 0.0002 | 31 | 0.0008 | 37 | 0.0015 | 18 | 0.0027 | 38 | 0.0016 | 39 | 0.0011 | 38 | 0.0027 | 28 | 0.0005 | 41 | 0.0024 | 37 |
| Precip 3 days prior | 0.0001 | 37 | 0.0002 | 31 | 0.0006 | 37 | 0.0008 | 33 | 0.0005 | 37 | 0.0005 | 34 | 0.0012 | 29 | 0.0002 | 35 | 0.0009 | 31 |
| Precip 3 months prior | 0.0006 | 21 | 0.0027 | 35 | 0.0037 | 6 | 0.0059 | 29 | 0.0040 | 32 | 0.0030 | 26 | 0.0059 | 21 | 0.0012 | 26 | 0.0060 | 24 |
| Precip 3 quarters prior | 0.0029 | 34 | 0.0074 | 38 | 0.0048 | 17 | 0.0061 | 22 | 0.0061 | 14 | 0.0045 | 16 | 0.0090 | 20 | 0.0017 | 29 | 0.0068 | 16 |
| Precip 3 weeks prior | 0.0003 | 19 | 0.0010 | 11 | 0.0016 | 26 | 0.0020 | 31 | 0.0016 | 19 | 0.0011 | 25 | 0.0032 | 22 | 0.0006 | 28 | 0.0022 | 27 |
| Deciduous 100m | 0.0000 | 54 | 0.0003 | 48 | 0.0002 | 54 | 0.0002 | 52 | 0.0000 | 54 | 0.0001 | 54 | 0.0000 | 54 | 0.0000 | 54 | 0.0013 | 48 |
| Deciduous 1000m | 0.0125 | 10 | 0.0003 | 47 | 0.0046 | 27 | 0.0019 | 45 | 0.0058 | 22 | 0.0013 | 44 | 0.0013 | 47 | 0.0000 | 53 | 0.0014 | 47 |
| Evergreen 100m | 0.0000 | 53 | 0.0000 | 54 | 0.0005 | 52 | 0.0005 | 51 | 0.0007 | 49 | 0.0006 | 50 | 0.0004 | 53 | 0.0012 | 40 | 0.0009 | 52 |
| Mixed forest 100m | 0.0001 | 51 | 0.0001 | 53 | 0.0007 | 49 | 0.0002 | 54 | 0.0058 | 21 | 0.0008 | 49 | 0.0007 | 52 | 0.0001 | 52 | 0.0003 | 54 |
| Mixed forest 1000m | 0.0005 | 43 | 0.0056 | 14 | 0.0039 | 33 | 0.0236 | 9 | 0.0020 | 43 | 0.0063 | 20 | 0.0016 | 46 | 0.0005 | 47 | 0.0041 | 40 |
| Forest 100m | 0.0001 | 47 | 0.0003 | 49 | 0.0059 | 21 | 0.0002 | 53 | 0.0011 | 48 | 0.0015 | 43 | 0.0008 | 51 | 0.0006 | 44 | 0.0010 | 49 |
| Forest 1000m | 0.0026 | 20 | 0.0157 | 5 | 0.0675 | 3 | 0.0069 | 28 | 0.0283 | 7 | 0.0140 | 15 | 0.0035 | 40 | 0.0562 | 2 | 0.0125 | 17 |
| Herbaceous 100m | 0.0016 | 22 | 0.0008 | 44 | 0.0030 | 43 | 0.0045 | 40 | 0.0030 | 42 | 0.0051 | 22 | 0.0025 | 44 | 0.0007 | 42 | 0.0023 | 45 |
| Herbaceous 1000m | 0.0044 | 15 | 0.0055 | 15 | 0.0102 | 16 | 0.0171 | 12 | 0.0148 | 9 | 0.0228 | 12 | 0.0117 | 18 | 0.0033 | 15 | 0.0156 | 10 |
| Shrubland 100m | 0.0050 | 13 | 0.0012 | 40 | 0.0058 | 23 | 0.0136 | 15 | 0.0017 | 44 | 0.0053 | 21 | 0.0025 | 45 | 0.0028 | 18 | 0.0044 | 38 |
| Shrubland 1000m | 0.0037 | 17 | 0.0075 | 10 | 0.0121 | 14 | 0.0296 | 6 | 0.0079 | 16 | 0.0242 | 10 | 0.0118 | 17 | 0.0070 | 9 | 0.0115 | 18 |
| Wetlands 100m | 0.0011 | 30 | 0.0001 | 51 | 0.0038 | 35 | 0.0149 | 14 | 0.0044 | 31 | 0.0042 | 27 | 0.0037 | 39 | 0.0027 | 19 | 0.0024 | 43 |
| Wetlands 1000m | 0.0035 | 18 | 0.0026 | 32 | 0.0188 | 8 | 0.0830 | 3 | 0.0359 | 4 | 0.0330 | 8 | 0.0326 | 9 | 0.0110 | 6 | 0.0219 | 8 |
| Low Developed 100m | 0.4335 | 1 | 0.0062 | 13 | 0.0063 | 19 | 0.0218 | 10 | 0.0138 | 12 | 0.0398 | 7 | 0.0086 | 23 | 0.0029 | 17 | 0.0106 | 19 |
| Low Developed 1000m | 0.0092 | 12 | 0.0291 | 3 | 0.0150 | 11 | 0.0276 | 8 | 0.0285 | 6 | 0.0849 | 4 | 0.0219 | 11 | 0.0073 | 7 | 0.0562 | 5 |
| Developed 100m | 0.0808 | 3 | 0.0042 | 18 | 0.0178 | 9 | 0.0290 | 7 | 0.0147 | 10 | 0.0153 | 14 | 0.0140 | 15 | 0.0046 | 11 | 0.0150 | 12 |
| Developed 1000m | 0.0325 | 6 | 0.0068 | 12 | 0.0140 | 12 | 0.2808 | 1 | 0.0936 | 2 | 0.0926 | 3 | 0.0768 | 5 | 0.0066 | 10 | 0.1165 | 3 |
