# Supplementary material for: Ecological drivers of dog heartworm transmission in California
Source: Parasit Vectors. 2022 Oct 23;15:388. doi: 10.1186/s13071-022-05526-x (PMC9590206; doi:10.1186/s13071-022-05526-x)
Supplement: Supplementary file 7 — Additional file 7: Table S5. Mean values of the ecological predictors for when a given vector species is absent (0) or present (1). Predictors that were ranked as among the top 10 most important, based on mean gain, for that species are denoted with a *. [file 13071_2022_5526_MOESM7_ESM.docx]

**Additional File 7**

**Table S5.** Mean values of the ecological predictors for when a given vector species is absent (0) or present (1). Predictors that were ranked as among the top 10 most important, based on mean gain, for that species are denoted with a *.

|  | *Ae. aegypti* | | *Ae. albopictus* | | *Ae. sierrensis* | | *Ae.*  *vexans* | | *An. freeborni* | | *Cs. incidens* | | *Cs. inornata* | | *Cx.*  *quinquefasciatus* | | *Cx.*  *tarsalis* | |
| --- | --- | --- | --- | --- | --- | --- | --- | --- | --- | --- | --- | --- | --- | --- | --- | --- | --- | --- |
| Absence/ Presence | 0 | 1 | 0 | 1 | 0 | 1 | 0 | 1 | 0 | 1 | 0 | 1 | 0 | 1 | 0 | 1 | 0 | 1 |
| Max temp 1 day prior | 28.93 | 32.22 | 29.21 | 29.31 | 29.25 | 28.12 | 29.15 | 31.37 | 29.08 | 30.92 | 29.99 | 26.52 | 29.46 | 24.33 | 28.63 | 29.61 | 28.17 * | 30.19 * |
| Max temp 1 week prior | 28.83 | 32.22 | 29.11 | 29.31 | 29.11 | 27.13 | 29.06 | 31.13 | 29.00 | 30.67 | 29.91 | 26.35 | 29.37 | 24.11 | 28.55 | 29.50 | 28.08 | 30.09 |
| Max temp 1 month prior | 28.78 | 32.24 | 29.07 | 29.29 | 28.75 | 25.52 | 29.02 | 31.16 | 28.96 | 30.59 | 29.88 | 26.30 | 29.33 | 24.14 | 28.54 | 29.44 | 28.07 | 30.02 |
| Max temp 1 quarter prior | 28.74 | 32.25 | 29.03 | 29.35 | 27.18 | 22.02 | 28.98 | 31.12 | 28.92 | 30.56 | 29.83 * | 26.27 * | 29.29 | 24.18 | 28.44 | 29.44 | 28.08 | 29.93 |
| Max temp 2 quarters prior | 28.45 | 32.27 | 28.76 | 29.39 | 21.52 | 15.25 | 28.70 | 31.05 | 28.66 | 30.17 | 29.62 | 25.81 | 29.00 * | 24.28 * | 28.12 | 29.21 | 27.92 | 29.56 |
| Max temp 3 quarters prior | 28.21 | 32.33 | 28.55 * | 29.24 * | 21.87 | 21.99 | 28.49 | 31.01 | 28.45 | 29.96 | 29.48 | 25.36 | 28.76 | 24.53 | 27.89 | 29.01 | 27.77 | 29.29 |
| Min temp 1 day prior | 28.29 | 32.28 | 28.62 | 29.26 | 14.37 | 11.33 | 28.56 | 31.03 | 28.52 | 30.02 | 29.52 | 25.53 | 28.84 * | 24.50 * | 27.96 | 29.08 | 27.81 | 29.39 |
| Min temp 1 month prior | 26.80 | 31.56 | 27.18 | 28.41 | 14.12 | 10.07 | 27.11 | 30.25 | 27.13 | 28.03 | 28.23 | 23.62 | 27.28 * | 25.43 * | 26.34 | 27.78 | 26.73 | 27.62 |
| Min temp 1 quarter prior | 24.72 * | 29.27 * | 25.10 | 25.93 | 12.98 * | 7.86 * | 25.02 | 28.34 | 25.10 | 25.10 | 26.10 | 21.66 | 25.06 | 25.88 | 24.09 | 25.81 | 25.04 | 25.16 |
| Diurnal temp 1 day prior | 12.46 | 16.18 | 12.76 | 14.94 | 14.89 | 16.79 | 12.74 | 13.77 | 12.85 | 11.58 | 13.29 | 10.99 | 12.84 | 11.37 | 11.50 | 13.66 | 13.01 | 12.55 |
| Diurnal temp 2 days prior | 8.30 | 10.30 | 8.46 | 9.20 | 14.80 | 16.37 | 8.45 | 9.08 | 8.61 | 6.35 | 8.72 | 7.59 | 8.29 | 11.81 | 7.37 | 9.22 | 9.26 | 7.70 |
| Diurnal temp 3 days prior | 8.87 | 7.32 | 8.76 | 6.48 | 14.82 | 16.10 | 8.77 | 7.86 | 8.93 | 6.13 | 8.39 | 9.95 | 8.63 | 10.84 | 7.64 | 9.51 | 9.49 | 8.04 |
| Diurnal temp 1 week prior | 0.08 | 0.02 | 0.07 | 0.01 | 14.81 | 16.16 | 0.07 | 0.08 | 0.07 | 0.08 | 0.07 | 0.09 | 0.07 | 0.15 | 0.09 | 0.06 | 0.09 | 0.06 |
| Diurnal temp 2 weeks prior | 0.09 | 0.02 | 0.08 | 0.03 | 14.67 | 15.62 | 0.08 | 0.09 | 0.08 | 0.09 | 0.07 | 0.11 | 0.08 | 0.16 | 0.10 | 0.07 | 0.09 | 0.07 |
| Diurnal temp 3 weeks prior | 0.09 | 0.03 | 0.08 | 0.02 | 14.59 | 15.26 | 0.08 | 0.09 | 0.08 | 0.10 | 0.08 | 0.11 | 0.08 | 0.18 | 0.10 | 0.07 | 0.10 | 0.07 |
| Diurnal temp 1 quarter prior | 0.41 | 0.13 | 0.39 | 0.16 | 14.20 | 14.15 | 0.39 | 0.42 | 0.39 | 0.40 | 0.36 | 0.50 | 0.37 | 0.75 | 0.44 | 0.36 | 0.45 | 0.33 |
| Diurnal temp 2 quarters prior | 0.47 | 0.16 | 0.44 | 0.19 | 12.85 | 11.65 | 0.44 | 0.47 | 0.44 | 0.44 | 0.40 | 0.57 | 0.42 | 0.82 | 0.49 | 0.40 | 0.49 | 0.39 |
| Diurnal temp 3 quarters prior | 0.50 | 0.18 | 0.48 | 0.21 | 13.07 | 14.52 | 0.47 | 0.49 | 0.47 | 0.48 | 0.43 | 0.64 | 0.46 | 0.83 | 0.54 | 0.43 | 0.52 | 0.43 |
| Precip 1 day prior | 26.60 | 31.04 | 26.96 | 27.87 | 0.07 | 0.13 | 26.90 | 29.87 | 26.92 | 27.72 | 27.95 | 23.61 | 27.06 | 25.27 | 26.13 | 27.56 | 26.53 | 27.39 |
| Precip 2 days prior | 21.09 | 23.20 | 21.27 | 20.56 | 0.08 | 0.16 | 21.21 | 23.40 | 21.39 | 19.49 | 21.83 | 19.32 | 21.04 | 25.47 | 20.06 | 22.11 | 21.86 | 20.70 |
| Precip 3 days prior | 22.05 | 19.92 | 21.89 | 18.43 | 0.08 | 0.19 | 21.86 | 22.15 | 22.06 | 19.29 | 21.64 | 22.68 | 21.72 | 24.73 | 20.71 | 22.69 | 22.51 | 21.27 |
| Precip 1 week prior | 14.03 | 16.66 | 14.24 | 15.54 | 0.38 | 0.73 | 14.24 | 14.35 | 14.30 | 13.41 | 14.57 | 13.13 | 14.48 | 9.80 | 13.01 | 15.10 | 14.10 | 14.39 |
| Precip 2 weeks prior | 14.02 | 16.71 | 14.24 | 15.52 | 0.42 | 0.91 | 14.24 | 14.42 | 14.30 | 13.41 | 14.58 | 13.10 | 14.47 | 9.87 | 13.03 | 15.09 | 14.12 | 14.36 |
| Precip 3 weeks prior | 13.97 | 16.75 | 14.19 | 15.71 | 0.45 | 1.06 | 14.19 | 14.37 | 14.26 | 13.34 | 14.54 | 13.02 | 14.43 | 9.85 | 12.98 | 15.05 | 14.10 | 14.29 |
| Precip 1 month prior | 13.94 | 16.74 | 14.16 | 15.65 | 1.23 | 2.43 | 14.17 | 14.35 | 14.23 | 13.32 | 14.51 | 12.99 | 14.40 | 9.89 | 12.95 | 15.02 | 14.08 | 14.26 |
| Precip 2 months prior | 13.80 | 16.90 | 14.05 | 15.86 | 1.60 | 3.36 | 14.05 | 14.40 | 14.12 | 13.15 | 14.46 | 12.69 | 14.26 | 10.12 | 12.82 | 14.92 | 14.02 | 14.10 |
| Precip 3 months prior | 13.65 | 17.02 | 13.92 | 15.83 | 2.04 | 3.93 | 13.92 | 14.49 | 13.99 | 13.05 | 14.38 | 12.38 | 14.11 | 10.47 | 12.69 | 14.80 | 13.93 | 13.93 |
| Precip 1 quarter prior | 13.69 | 16.95 | 13.94 | 15.82 | 2.86 * | 4.79 * | 13.94 | 14.44 | 14.02 | 13.05 | 14.38 | 12.48 | 14.14 | 10.37 | 12.71 | 14.83 | 13.95 | 13.96 |
| Precip 2 quarters prior | 12.63 | 16.64 | 12.95 | 15.40 | 121.85 | 392.26 | 12.94 | 14.09 | 13.05 | 11.79 | 13.53 | 11.02 | 13.04 | 11.56 | 11.66 | 13.87 | 13.20 | 12.74 |
| Precip 3 quarters prior | 11.07 | 14.95 | 11.38 | 13.60 | 117.98 | 215.48 | 11.35 | 12.79 | 11.50 | 9.90 | 11.95 | 9.47 | 11.35 | 12.19 | 10.11 | 12.28 | 11.87 | 10.93 |
| Deciduous 100m | 1.74 | 0.88 | 1.68 | 0.95 | 0.00 | 0.01 | 1.67 | 1.67 | 1.66 | 1.78 | 1.51 | 2.22 | 1.65 | 2.09 | 1.86 | 1.54 | 1.68 | 1.66 |
| Deciduous 1000m | 14.56 * | 15.32 * | 14.63 | 13.41 | 0.00 | 0.01 | 14.57 | 16.52 | 14.46 | 16.91 | 15.10 | 12.98 | 14.65 | 14.06 | 15.20 | 14.21 | 13.84 | 15.36 |
| Evergreen 100m | 2.19 | 1.33 | 2.12 | 1.44 | 0.00 | 0.05 | 2.12 | 1.98 | 2.09 | 2.49 | 1.93 | 2.75 | 2.12 | 2.02 | 2.42 | 1.90 | 2.01 | 2.22 |
| Mixed forest 100m | 3.03 | 1.92 | 2.94 | 2.18 | 0.00 | 0.03 | 2.94 | 2.97 | 2.92 | 3.18 | 2.74 | 3.63 | 2.92 | 3.40 | 3.20 | 2.76 | 2.93 | 2.95 |
| Mixed forest 1000m | 14.60 | 15.33 | 14.67 | 13.44 | 0.00 | 0.03 | 14.61 * | 16.59 * | 14.50 | 16.97 | 15.13 | 13.05 | 14.69 | 14.12 | 15.25 | 14.25 | 13.86 | 15.42 |
| Forest 100m | 1.34 | 0.57 | 1.28 | 0.70 | 0.00 | 0.08 | 1.28 | 1.39 | 1.28 | 1.25 | 1.18 | 1.63 | 1.24 | 2.07 | 1.38 | 1.20 | 1.38 | 1.18 |
| Forest 1000m | 14.80 | 15.50 | 14.87 * | 13.70 * | 0.01 * | 0.11 * | 14.81 | 16.77 | 14.69 * | 17.24 * | 15.32 | 13.28 | 14.89 | 14.29 | 15.50 * | 14.42 * | 14.01 | 15.67 |
| Herbaceous 100m | 14.82 | 15.49 | 14.88 | 13.58 | 0.03 | 0.09 | 14.82 | 16.78 | 14.70 | 17.25 | 15.34 | 13.28 | 14.90 | 14.28 | 15.56 | 14.40 | 13.97 | 15.73 |
| Herbaceous 1000m | 12.79 | 12.90 | 12.81 | 11.36 | 0.04 | 0.15 | 12.76 | 14.32 | 12.78 * | 13.13 * | 13.11 | 11.74 | 12.76 | 13.66 | 12.69 | 12.88 | 12.60 * | 12.99 * |
| Shrubland 100m | 14.81 | 15.52 | 14.87 | 13.79 | 0.03 | 0.15 | 14.82 | 16.71 | 14.70 | 17.26 | 15.34 | 13.25 | 14.90 | 14.24 | 15.51 | 14.42 | 13.96 | 15.73 |
| Shrubland 1000m | 14.14 | 14.86 | 14.21 * | 12.92 * | 0.04 | 0.12 | 14.15 * | 16.10 * | 14.06 | 16.14 | 14.66 * | 12.62 * | 14.22 | 13.90 | 14.64 * | 13.90 * | 13.52 | 14.84 |
| Wetlands 100m | 136.28 | 93.27 | 132.65 | 147.65 | 0.03 | 0.06 | 133.08 | 119.59 | 127.13 | 212.03 | 118.07 | 182.75 | 135.23 | 85.65 | 187.50 | 94.41 | 113.87 | 150.73 |
| Wetlands 1000m | 14.65 | 15.37 | 14.71 | 13.53 | 0.02 * | 0.06 * | 14.66 * | 16.65 * | 14.54 * | 17.01 * | 15.17 * | 13.12 * | 14.73 * | 14.16 * | 15.31 * | 14.29 * | 13.91 * | 15.46 * |
| Low Developed 100m | 121.57* | 125.71* | 121.37 | 213.71 | 0.32 | 0.32 | 121.97 * | 119.69 * | 115.83 | 207.84 | 120.14 * | 127.95 * | 123.64 | 89.30 | 168.70 | 89.17 | 105.46 | 137.59 |
| Low Developed 1000m | 14.16 | 14.93 | 14.23 * | 13.01 * | 0.23 | 0.22 | 14.17 * | 16.15 * | 14.08 * | 16.24 * | 14.70 * | 12.60 * | 14.24 | 13.87 | 14.68 * | 13.91 * | 13.53 * | 14.88 * |
| Developed 100m | 14.91* | 15.56 * | 14.97 | 13.78 | 0.40 * | 0.10 * | 14.91 * | 17.02 * | 14.78 * | 17.51* | 15.42 | 13.39 | 14.99 | 14.53 | 15.62 | 14.51 | 14.07 | 15.81 |
| Developed 1000m | 13.66 * | 14.32 * | 13.72 | 12.32 | 0.41 | 0.14 | 13.66 * | 15.55 * | 13.61 * | 15.19 * | 14.15 * | 12.20 * | 13.71 * | 13.70 * | 13.97 * | 13.53 * | 13.17 * | 14.22 * |
